# Beyond Risk Scores: Translating PAH Risk Assessment into Clinical Benefit. Comment on Cortes-Telles et al. Sequential Add-On Therapy Modifies Mortality Risk Stratification in Group 1.4 Pulmonary Arterial Hypertension: A Real-World, Single-Center Retrospective Cohort Study from Mexico. *J. Clin. Med*. 2026, *15*, 4924

**DOI:** 10.3390/jcm15186969

**Published:** 2026-09-09

**Authors:** Ilma Nascimento, Caio Júlio César dos Santos Fernandes

**Affiliations:** Pulmonary Circulation Unit, Heart Institute (InCor), Hospital das Clínicas HCFMUSP, Faculdade de Medicina, Universidade de São Paulo, São Paulo 05403-900, Brazil

Cortes-Telles et al. report changes in risk classification after sequential add-on therapy with macitentan and sildenafil in 25 patients with Group 1.4 pulmonary arterial hypertension (PAH) treated at a referral center in Mexico [1]. Beyond providing regional data from a population under-represented in clinical trials, the study highlights a broader challenge in resource-constrained settings: identifying residual risk does not necessarily ensure that the healthcare system can modify it.

After 24 weeks, the proportion of patients classified as low risk increased from 19% to 43% according to COMPERA 2.0 and from 40% to 68% according to REVEAL Lite 2 [1]. Brain natriuretic peptide levels and World Health Organization functional class also improved. In contrast, the 6 min walk distance remained essentially unchanged, and the TAPSE/systolic pulmonary artery pressure ratio did not improve significantly. These findings illustrate that movement toward a lower predicted mortality category may not always be accompanied by parallel improvement across all functional or right ventricular measures.

This distinction is increasingly relevant as the meaning of “low risk” evolves. Fauvel et al. recently argued that short-term mortality alone may be insufficient to define low-risk PAH and proposed incorporating clinical worsening and longer-term mortality into a more demanding construct [2]. Complementary assessment of right ventricular function may provide additional prognostic information beyond conventional clinical and functional variables [3]. More broadly, prognosis in PAH is inherently multidimensional and reflects functional capacity, biomarkers, right ventricular adaptation, hemodynamic impairment, and other disease- and patient-related factors. Emerging prognostic markers, including circulating biomarkers, may provide additional information, but are more appropriately considered complementary to rather than substitutes for integrated risk assessment [4]. Risk scores remain essential, but their clinical significance ultimately depends on whether reclassification is accompanied by durable reductions in morbidity, hospitalization, symptom burden, and functional limitation.

The clinical value of simplified risk tools should also be interpreted according to the population in which they are applied. Adult-derived tools such as COMPERA 2.0 and REVEAL Lite 2 cannot be assumed to have equivalent performance or thresholds in pediatric PAH. Recent pediatric data underscore the need for age-specific validation and adaptation of prognostic models before these tools are incorporated into treatment decisions in children [5].

The study by Cortes-Telles et al. also reports intermittent drug shortages that limited continuous treatment access and longer-term follow-up [1]. This should not be regarded solely as a methodological limitation. It is a clinically meaningful finding that reflects the context in which risk-guided care must be implemented. Although intermittent drug shortages were directly documented in the cohort studied by Cortes-Telles et al., broader barriers involving access to specialized care, therapeutic availability, referral pathways, and the implementation of guideline-recommended management have been described separately across Latin America [6]. Thus, risk assessment may identify the need for treatment escalation without ensuring that escalation is available, initiated, or sustained.

Treatment continuity is especially important because reduced adherence and treatment interruption in PAH have been associated with hospitalization, disease progression, and mortality [7]. Real-world evaluations of treatment response should therefore consider not only the prescribed regimen and resulting risk category, but also medication availability, continuity of exposure, whether recommended treatment escalation was actually initiated, and the capacity of the healthcare system to act on residual risk.

More broadly, clinical information becomes actionable only when it can trigger an appropriate response [8]. Clinical decision support systems may facilitate this translation by organizing longitudinal information, highlighting changes in risk, and prompting reassessment when predefined clinical thresholds are reached. However, their value ultimately depends on whether the recommended response can be implemented in practice. Applied to PAH, contemporary consensus emphasizes risk assessment as a dynamic process intended to inform treatment decisions throughout the disease course rather than as a one-time classification exercise [9]. Accordingly, dynamic risk assessment may be conceptualized as a continuum: identification of risk, determination of whether treatment escalation is indicated, access to the recommended therapy, treatment initiation, continuity of exposure, and subsequent assessment of meaningful patient-level response. This perspective is consistent with the increasing incorporation of pragmatic and implementation-related questions alongside conventional efficacy endpoints in PAH research [10].

Future Latin American studies should therefore assess both risk transitions and the implementation pathways surrounding them. Simplified tools such as COMPERA 2.0 and REVEAL Lite 2 may be especially useful in resource-constrained environments, but their clinical impact depends on whether identified risk can be translated into timely and sustained treatment. Registries and real-world cohorts should report access-related measures alongside risk transitions, including time to treatment escalation, medication availability, the duration of treatment interruptions, whether recommended escalation was initiated, and the time between risk identification and actual treatment implementation. Such data would help distinguish favorable prognostic reclassification from improvement that is both clinically meaningful and sustainable in routine practice.

## Data Availability

No new data were created or analyzed in this study. Data sharing is not applicable to this article.
